# The declining occurrence of moose (*Alces alces*) at the southernmost edge of its range raise conservation concerns

**DOI:** 10.1002/ece3.7441

**Published:** 2021-03-30

**Authors:** Tomáš Janík, Wibke Peters, Martin Šálek, Dušan Romportl, Miloslav Jirků, Thomas Engleder, Martin Ernst, Jiří Neudert, Marco Heurich

**Affiliations:** ^1^ Faculty of Science Department of Physical Geography and Geoecology Charles University Praha Czechia; ^2^ Department of Spatial Ecology Silva Tarouca Research Institute for Landscape and Ornamental Gardening Průhonice Czechia; ^3^ Department of Visitor Management and National Park Monitoring Bavarian Forest National Park Grafenau Germany; ^4^ Bavarian State Institute of Forestry Freising Germany; ^5^ Czech Academy of Sciences Institute of Vertebrate Biology Brno Czechia; ^6^ Faculty of Environmental Sciences Czech University of Life Sciences Prague Praha Czechia; ^7^ Biology Centre of the Czech Academy of Sciences Institute of Parasitology České Budějovice Czechia; ^8^ Green Heart of Europe Haslach AT Austria; ^9^ Faculty of Forestry and Wood Technology Department of Forest Protection and Wildlife Management Mendel University in Brno Brno Czechia; ^10^ Administration of Třeboňsko Protected Landscape Area and Biospheric Reservation Třeboň Czechia; ^11^ Faculty of Environment and Natural Resources Chair of Wildlife Ecology and Management University of Freiburg Freiburg Germany; ^12^ Inland Norway University of Applied Science Institute for Forest and Wildlife Management Koppang Norway

**Keywords:** Bohemian Forest Ecosystem, Habitat suitability modelling, Moose (alces alces)

## Abstract

The border region between Austria, the Czech Republic, and Germany harbors the most south‐western occurrence of moose in continental Europe. The population originated in Poland, where moose survived, immigrated from former Soviet Union or were reintroduced after the Second World War expanded west‐ and southwards. In recent years, the distribution of the nonetheless small Central European population seems to have declined, necessitating an evaluation of its current status. In this study, existing datasets of moose observations from 1958 to 2019 collected in the three countries were combined to create a database totaling 771 records (observations and deaths). The database was then used to analyze the following: (a) changes in moose distribution, (b) the most important mortality factors, and (c) the availability of suitable habitat as determined using a maximum entropy approach. The results showed a progressive increase in the number of moose observations after 1958, with peaks in the 1990s and around 2010, followed by a relatively steep drop after 2013. Mortality within the moose population was mostly due to human interactions, including 13 deadly wildlife‐vehicle collisions, particularly on minor roads, and four animals that were either legally culled or poached. Our habitat model suggested that higher altitudes (ca. 700–1,000 m a.s.l.), especially those offering wetlands, broad‐leaved forests and natural grasslands, are the preferred habitats of moose whereas steep slopes and areas of human activity are avoided. The habitat model also revealed the availability of large core areas of suitable habitat beyond the current distribution, suggesting that habitat was not the limiting factor explaining the moose distribution in the study area. Our findings call for immediate transboundary conservation measures to sustain the moose population, such as those aimed at preventing wildlife‐vehicle collisions and illegal killings. Infrastructure planning and development activities must take into account the habitat requirements of moose.

## Graphical abstract

Moose at the southernmost edge of its distribution in Europe deserve attention and conservation. We combined data from three countries to analyze history and current distribution, opportunities and threats for this population.

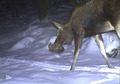

## 1 INTRODUCTION

In several parts of the world, particularly in developing countries, populations of large herbivores are decreasing, mainly due to overexploitation and habitat loss (Apollonio et al., 2010, 2017; Bragina et al., 2018; Linnel et al., 2020). In Europe and North America, by contrast, large herbivore populations have generally increased over the last several decades (Apollonio et al., 2010, 2017; Bragina et al., 2018), primarily due to changes in hunting management, better habitat quality as a consequence of improved land‐use practices (e.g., change to multi‐species forestry) and land abandonment in rural areas (Boitani & Linnel, 2015). Nonetheless, in recent years, population declines of some species of large herbivores have been reported also in Europe (Loison et al., 2003, Putman et al., 2011). Changes in large herbivore populations have important implications, as in addition to influencing vegetation composition and patch heterogeneity these animals are important drivers of ecosystem processes (Hobbs, 1996; Ripple et al., 2015, 2016).

**FIGURE 1:**
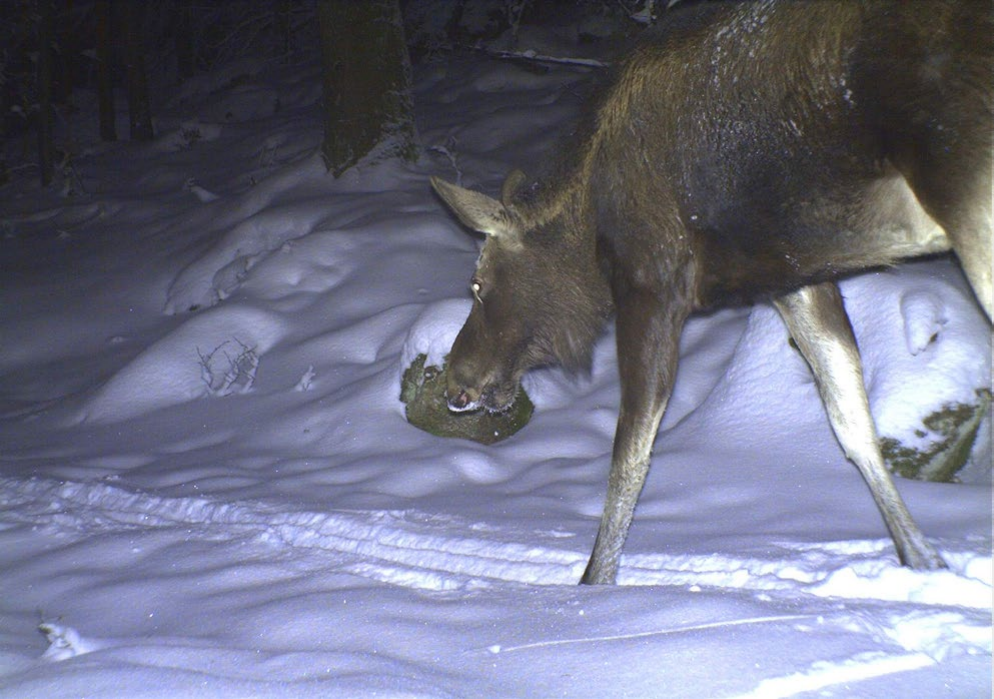
Moose (*Alces alces* in our study area, photo by Thomas Engleder)

Moose (*Alces alces*, Figure 1) is the largest cervid and, along with the European bison (*Bison bonasus*), the largest mammal native to Europe (Niedziałkowska, 2017; Schmölcke & Zachos, 2005). During the Holocene, moose covered almost all of Europe (Niedziałkowska, 2017), but in the Middle Ages, populations in central, western, and southern Europe began to decline significantly, mainly due to human‐caused mortality (Kyselý, 2005; Niedziałkowska, 2017; Schmölcke & Zachos, 2005) but later also as a consequence of increased human activity such as infrastructure development, which decreased forest cover and caused landscape fragmentation (Niedziałkowska et al., 2014; Schmölcke & Zachos, 2005). By the beginning of the 20th century, the distribution of moose had reached its smallest extent (Niedziałkowska, 2017), limited to Fennoscandia, parts of Poland, the Baltic states, Belarus, and Russia (Niedziałkowska et al., 2014). Caused by immigration from the former Soviet Union and the expansion of the relict population in the Biebrza National Park amended by reintroductions in Poland, the population has gradually increased since end of World War II (Niedziałkowska, 2017; Świsłocka et al., 2013). Moose can now be found from Scandinavia and Eastern Poland, across the Baltic states to Belarus and Ukraine, and to Russia, up to the Yenisei river (Bauer & Nygrén, 1999; Corbet, 1979; Grubb, 2005). The southernmost range is in Central Europe (Romportl et al., 2017; Schmölcke & Zachos, 2005), as southern populations became established in the south‐western region of the Czech Republic. After the fall of the Iron Curtain and into the 1990s, moose dispersed to Austria and Germany (Mrlík, 1995; Schönfeld, 2009). However, since 2010, this south‐westernmost European moose population has progressively decreased in size (Romportl et al., 2017).

Moose prefer early seral forest stages with an abundance of deciduous shrubs that provide browsing (Courtois et al., 2002), but they also seek cover in closed forests (e.g., mature coniferous forest) (Beest et al., 2012; Melin et al., 2016). By adapting their temporal and spatial habitat use to changing environmental conditions, moose ensure the fulfillment of their physiological needs (Ball et al., 2001; Dussault et al., 2005). In addition, because moose are sensitive to high temperatures, they rely on a combination of lakes or ponds (Bjørneraas et al., 2011; Dussault et al., 2004) and closed or mature forests to mitigate heat stress (Borowik et al., 2020; Melin et al., 2016). These water bodies also provide a nutrient‐rich supply of food (Borowik et al., 2018; Tomek, 1977). In winter, moose prefer regenerating and young forests, which offer both woody browsing and shelter (Beest et al., 2012; McNicol & Gilbert, 1980; Melin et al., 2016). Moose habitats in Central Europe often comprise forested landscapes (Schönfeld, 2009), with a mosaic of pastures and a diverse landscape structure (Romportl et al., 2017). In Poland, moose inhabit lowland forests with shrubs but also bogs as well as mire habitats with sedge‐moss and reed communities (Borowik et al., 2018; Tomek, 1977). Overall, moose are flexible in their habitat use and will adapt to different conditions as long as abundant foraging opportunities and forested shelters are present (Bjørneraas et al., 2011).

Moose are highly mobile animals and their home ranges are large, between 10 and 60 km^2^ depending on the age, sex and reproductive status of the animal, and environmental characteristics such as forage availability and climatic conditions (Beest et al., 2011; Cederlund & Sand, 1994; Murray et al., 2012). Migrations are common in seasonal habitats (Ball et al., 2001; Rolandsen et al., 2017). In Scandinavia, for example, moose spend the summer at higher elevations because of better food quality and lower temperatures and then migrate in autumn, once snow limits their movement and food accessibility, to reach lower elevations (Andersen, 1991; Bunnefeld et al., 2011; Melin et al., 2013, 2016). Similarly, moose can disperse over long distances, for example, from central Poland to southern Bohemia (Niedziałkowska et al., 2014), which has important implications for their potential range expansion. However, during these journeys, moose may block roads and otherwise interfere with infrastructure, such that they are notoriously susceptible to traffic‐associated mortality (e.g., Seiler, 2005). Compared to moose distribution in Scandinavia and Canada, Central Europe has a much higher road density, and the absence of suitable crossing points on motorways and main roads hinders the safe movements of large animals, including moose (Strnad et al., 2012). Central European motorways extend over several thousand kilometers, and traffic intensity has substantially increased. Consequently, wildlife‐vehicle collisions have become an important cause of death for large herbivores (Bragina et al., 2018; Mrlík, 1995), and large patches of suitable habitat for moose have become increasingly isolated (Ree et al., 2015).

In this study, we examined moose populations within the transboundary region of Austria, the Czech Republic, and Germany. Although moose are listed as endangered and are fully protected by conservation laws in the Czech Republic as well as by hunting laws in Bavaria and Austria, the size and distribution of moose in the study region have been stagnant and possibly declining (Romportl et al., 2017; Schönfeld, 2009). Thus, in this study, to obtain an accurate picture of the history and status of the moose population, we collected all available occurrence data from the region to (a) evaluate changes in moose distribution in the study area; (b) identify the main causes of mortality; and (c) analyze habitat selection to model the extent of suitable habitat. We expected that landscapes with water bodies, wetlands, and a heterogeneous mosaic of shrubs, meadows, and forests provide suitable habitat for the moose population (Beest et al., 2012; Borowik et al., 2018, 2020; Courtois et al., 2002; Melin et al., 2016; Romportl et al., 2017; Tomek, 1977), while anthropogenic structures would reduce the amount and connectivity of available habitat (Bragina et al., 2018; Mrlík, 1995; Ree et al., 2015; Seiler, 2005; Strnad et al., 2012).

## 2 MATERIAL AND METHODS

### 2.1 Study area

**FIGURE 2:**
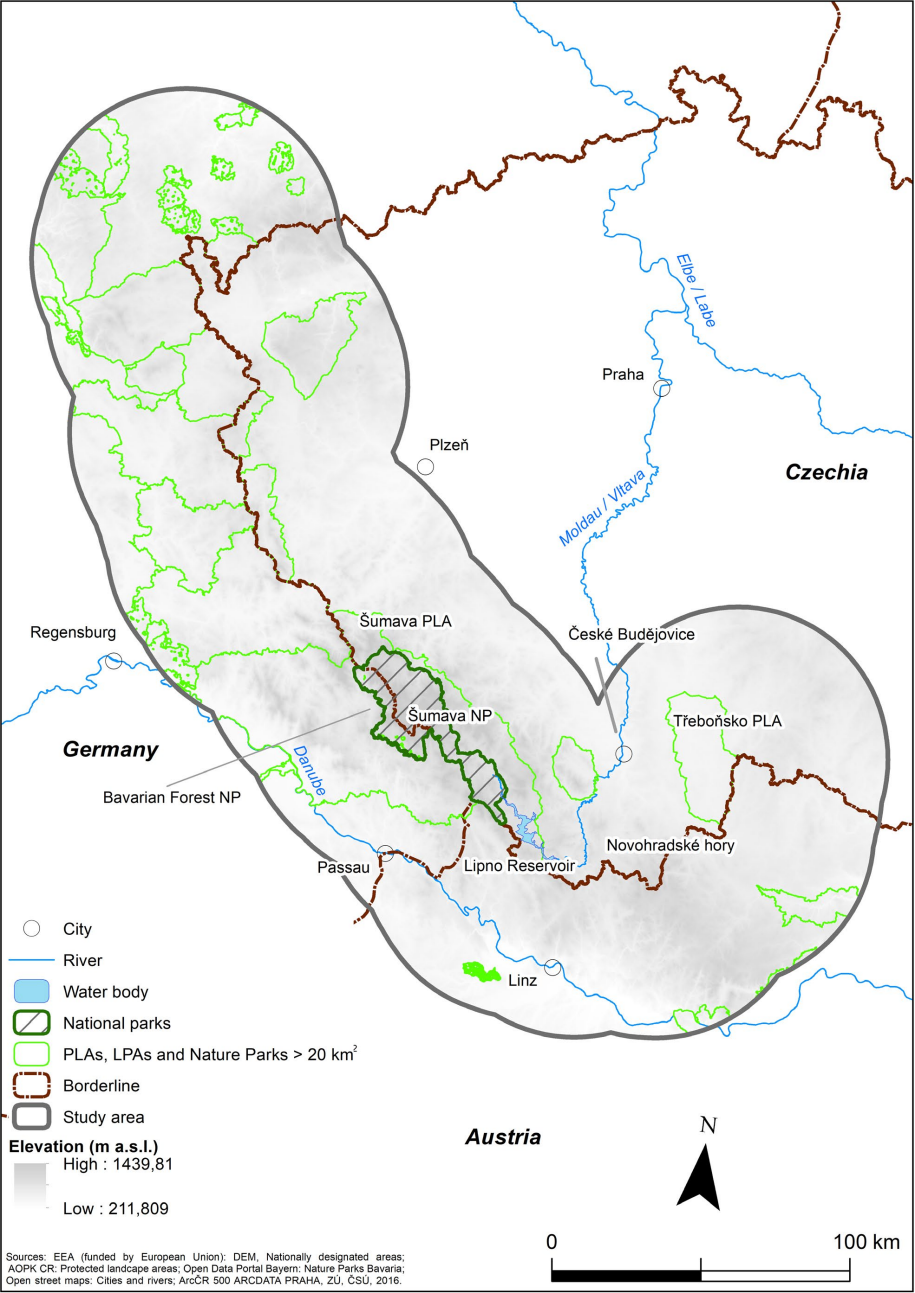
The study area was situated within the 50 km buffer along the Austrian–German–Czech border. Large protected areas and major geographic features are described in the text

The border of the study area consisted of a 50‐km buffer along the borders of Austria, the Czech Republic, and Germany, between 48.1 N and 51.0 N (44,100 km^2^; Figure 2) and covering the core area of moose distribution in Central Europe (Homolka, 1998). Forests comprise 40% (85% coniferous), arable land 27.2%, pastures 17.2%, heterogeneous agricultural areas 8%, and artificial surfaces 5% of the study area. From the south‐west and south, the study area is naturally bordered by the Danube valley, which includes barriers formed by the Danube itself but also by artificial structures. On the Czech side, the study area is delimited by larger cities (Plzeň and České Budějovice) and landscape with increasing human population density. Šumava National Park (SNP) and Bavarian Forest National Park (BNP) are located in the central part of the study area, together forming one of the largest protected forested areas in Central Europe (Křenová & Hruška, 2012).

The core area is created by mountain ranges along the Austrian, Czech, and German borders, with the highest altitudes occurring in the Bohemian Forest, that is, the Šumava Mts. Forested mountain ranges and highland plateaus with mosaics of peat bogs and extensively used grasslands create the typical landscape of the central SNP and the mountain ranges of the region in general (Janík & Romportl, 2016; Spitzer & Bufková, 2008; Wölfl et al., 2001). The elevation varies between 210 and 1.456 m a.s.l., and the mean annual temperature in the study area ranges from 3 to 9°C (Heurich et al., 2010; Tolasz et al., 2007).

The eastern edge of the study area is in the Třeboňsko Protected Landscape Area (PLA) and adjacent areas (Třeboňsko hereafter), a flat sedimentary basin with acidic soils and an elevation ranging between 400 and 550 m a. s. l. Land cover is mainly characterized by forests, with a smaller amount of agricultural land (e.g., meadows and pastures), human settlements, and various wetland habitats (e.g., peat bogs), including extensive systems of man‐made water bodies, such as fishponds and channels (Hanák et al., 2006).

### 2.2 Moose distribution and mortality

Data on moose occurrence were gathered from local stakeholders, regional administrations, hunters (questionnaires filled out by hunting organizations for the Ministry of Environment of the Czech Republic, local hunters’ journals, and web pages), scientists (including local scientific journals and webpages), the public and through an extensive literature search of zoological databases (GeneDBase, zoological records, Web of Science; Czech National Database of Fauna‐Flora Records [NDOP], AOPK ČR, 2020). There has been no continuous monitoring on moose throughout the study area. Therefore, we collected all the data, which are in different quality and from mentioned sources. All of the acquired information was transferred to the database, and three categories were distinguished based on the SCALP classification scheme (Molinari‐Jobin et al., 2012): C1: undoubtful records (dead animals, photograph documentation, camera traps, and genetic records), C2: confirmed records (moose tracks, scats, or assessable field signs verified by experts), and C3: unconfirmed data (observations that could not be confirmed by experts).

To assess long‐term changes, occurrence data were divided into four periods: 1958–1989; 1990–1999; 2000–2009; and 2010–2019. The 30‐year‐long first period covered the initial phase of moose establishment in the study area, from the very first occurrences to the development of a breeding population and ending with the disappearance of a major migration barrier for large mammals, that is, the fall of the border fence (Iron Curtain) in 1989/1990, which resulted from significant political and socio‐economic changes. The following three periods represent moose occurrence patterns during the three decades when movement of the moose population was not hindered by the border fence. The analyses and visualizations of this study were based on 10 km × 10 km squares. Moose occurrence during each period was classified as (a) *reproduction*, when the presence of female(s) with a calf(calves) within an individual period was recorded; (b) *regular occurrence*, consisting of >5 occurrences within a single period; and (c) *irregular occurrence*, when ≤5 occurrences within an individual period were recorded (see also Wölfl et al., 2001). The cause of mortality was categorized as (a) wildlife‐vehicle collision, (b) legal culling, (c) poaching, and (d) unknown.

### 2.3 Environmental data and habitat preferences

Land cover variables were obtained from Corine Land Cover (CLC) for the years 1990, 2000, 2006, and 2012 (European Environment Agency, 2013a). Eleven land cover categories were defined: (a) artificial surfaces, (b) arable land, (c) permanent crops, (d) pastures, (e) heterogeneous agricultural areas, (f) broad‐leaved forest, (g) coniferous forest, (h) mixed forest, (i) natural grassland and shrubs, (j) wetlands, and (k) waterbodies. Road density (length of road per area) for each pixel and the distances to settlements, roads, and forests were calculated based on Corine and Open Street Map (OSM) data. Terrain characteristics (i.e., elevation and slope) were obtained from a digital elevation model (DEM) provided by the European Environmental Agency (25‐m pixel resolution) (European Environment Agency,  2013b). Environmental variables were rasterized according to the resolution of the DEM (25 m). Temperature was excluded from further analysis because of its correlation (more than 0.65) with DEM (Dormann et al., 2013). Finally, DEM, land cover, slope, road density, and distances to settlements, roads, and forests were included as independent variables. All GIS computations were done using ArcGIS 10.5 software (ESRI, 2016). Maxent 3.4.1 software (Elith et al., 2011) was used for habitat modeling with presence‐only data. The contributions of the variables, the response curves, and their importance were computed. The Maxent approach is based on maximum entropy in estimates of a target probability of species´ distribution (Phillips et al., 2006). In this study, the model was implemented using 10,000 background points, evaluated based on 500 iterations and 10% of the data and assessed using the AUC (area under curve) (Phillips et al., 2006).

We calculated several MaxEnt models for each period with corresponding land cover (1958–1989 = CLC1990, 1990–1999 = CLC2000, 2000–2009 = CLC2006, and 2010–2019 = CLC2012; there is also CLC 2018, but more records were collected between 2010 and 2015, therefore, we used CLC2012). In the final habitat suitability model, we used all moose occurrence data and the land cover data from 2012. Furthermore, we validated the model only with records from C1 and C2 categories.

Additionally, we evaluated differences in the distances of the moose records from key land cover categories: artificial surfaces as potential barrier and threat, broad‐leaved forest as a preferred habitat for foraging and hiding, natural grassland and shrubs for foraging, wetlands, and waterbodies as habitats for hiding and avoiding heat stress (Borowik et al., 2018, 2020; Beest et al., 2012; Courtois et al., 2002; Melin et al., 2016; Romportl et al., 2017; Tomek, 1977) for each time period. We tested data for normality and used ANOVA and Kruskal–Wallis test depending on normality of the data in R software (R Core Team, 2019).

## 3 RESULTS

### 3.1 Moose distribution

**FIGURE 3:**
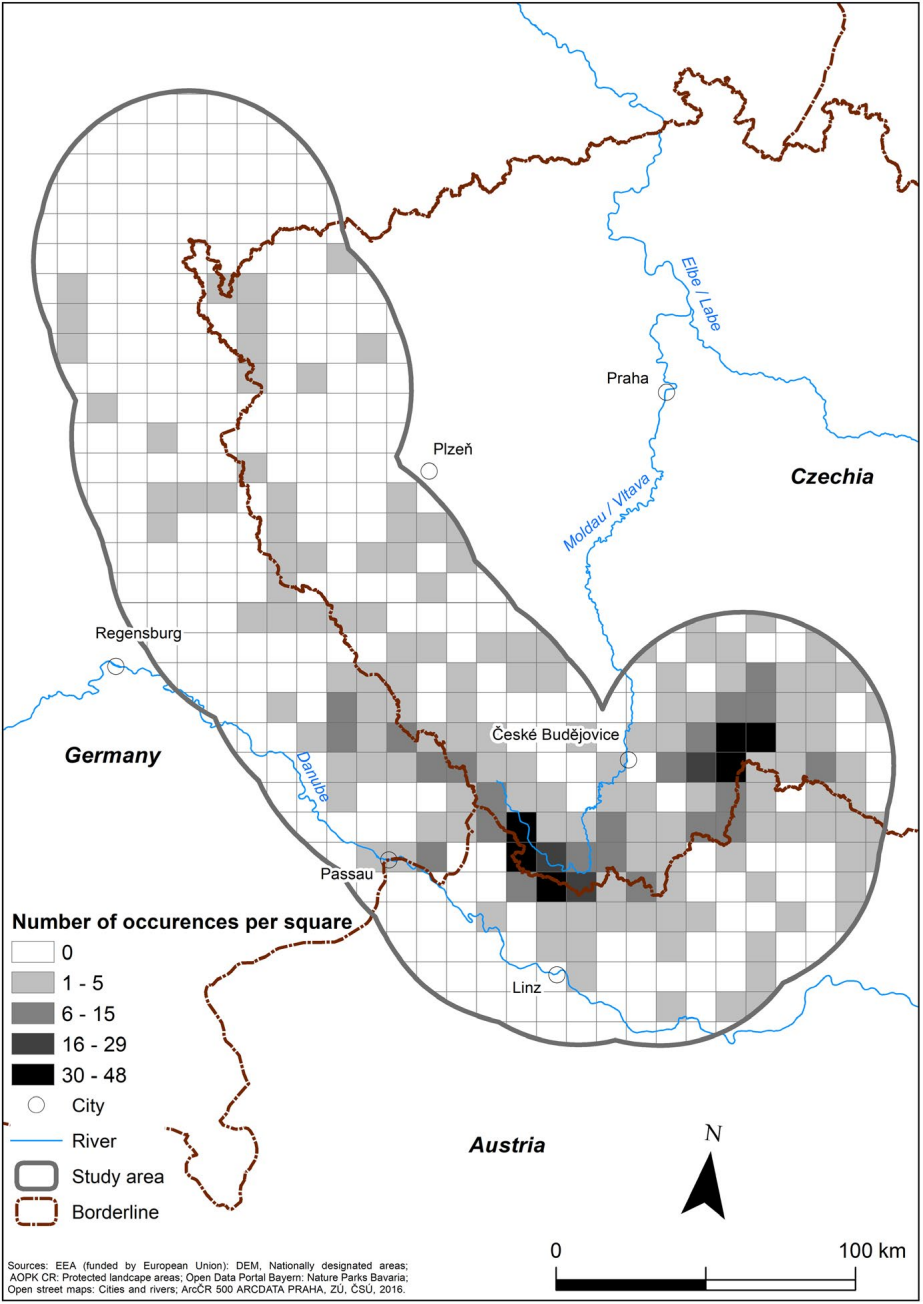
Moose distribution in the study area during the study period (1958–2019)

**FIGURE 4:**
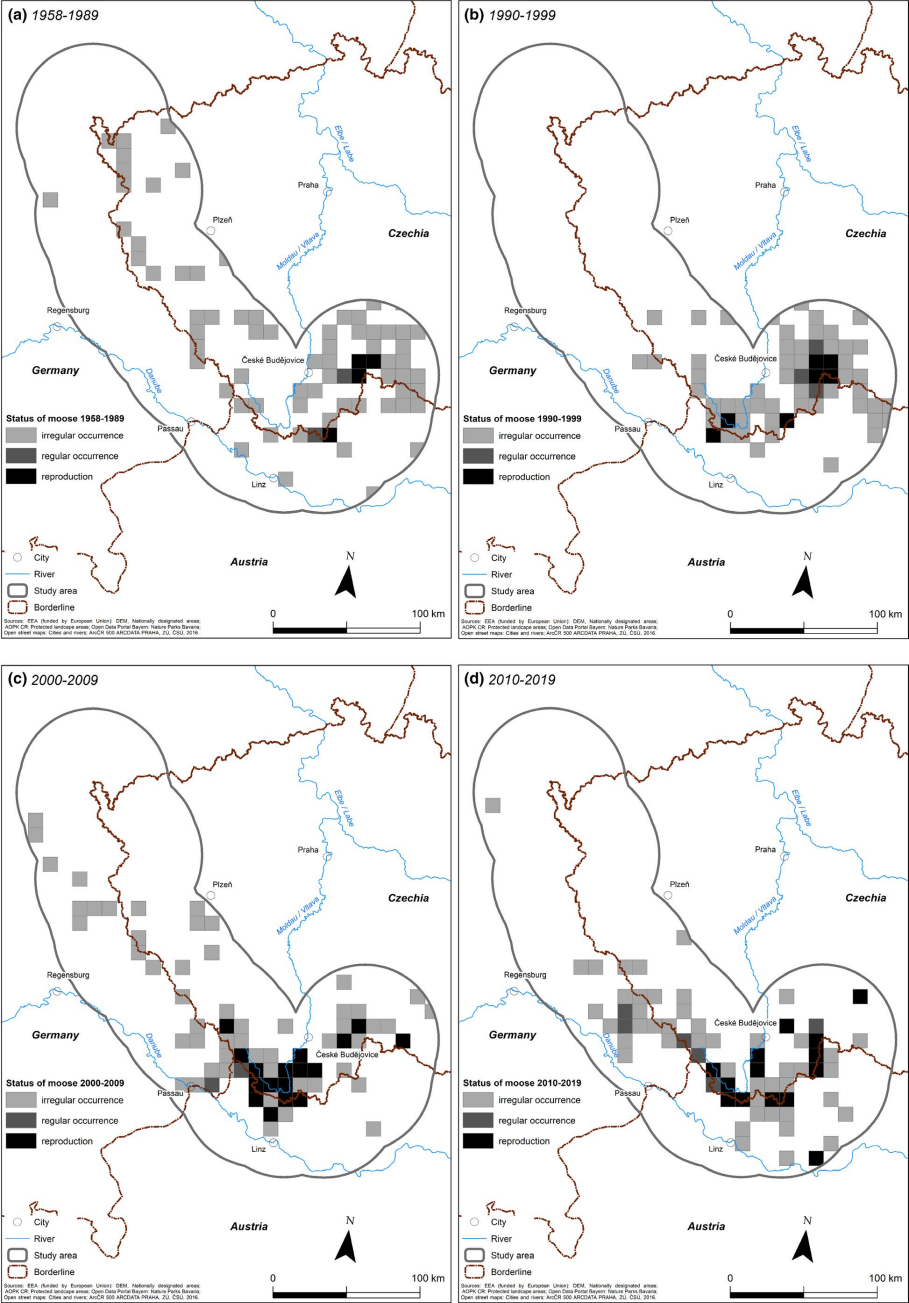
Changes in moose occurrence along the Austrian–German–Czech border during: (a) 1958–1989; (b) 1990–1999; (c) 2000–2009; and (d) 2010–2019

**FIGURE 5:**
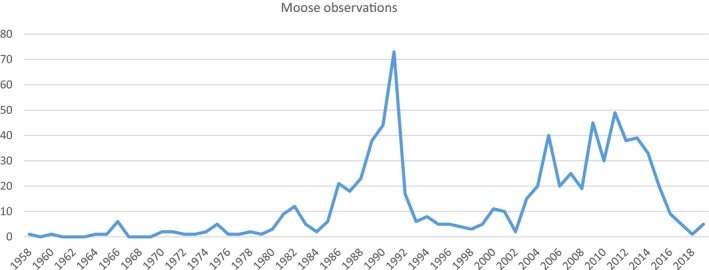
Development of moose observations between 1958 and 2019

For the period between 1958 and 2019, there were 771 moose observations: 165 during the first period (1958–1989), 170 during the second period (1990–1999), 207 during the third period (2000–2009), and 229 during the fourth period (2010–2019) (Table 1, Figures 3, 4, 5). The distribution of these observations suggested a spatial shift in moose occurrence (Figure 4); the highest numbers of observations were around years 1990 and 2010 (Figure 5).

**TABLE 1:** Numbers of records for each country, period, and class of quality data

	Austria				Czechia				Germany				Total			
Period	Total	C1	C2	C3	Total	C1	C2	C3	Total	C1	C2	C3	Total	C1	C2	C3
1958–1989	27	2	1	24	129	34	7	88	9	1	6	2	165	37	14	114
1990–1999	29	4	9	16	138	16	2	120	3	0	3	0	170	20	14	136
2000–2009	33	4	3	26	134	22	21	91	40	7	5	28	207	33	29	145
2010–2019	39	19	6	14	146	78	53	15	44	17	9	18	229	114	68	47

During the first period (1958–1989), moose were observed in 75 out of 502 squares (Figure 4). Moose distribution was characterized by an irregular occurrence, especially across the parts of the study area located in the Czech Republic. Specifically, in the core area of moose distribution in Třeboňsko PLA, reproduction was confirmed in three squares (Figure 4a). During the second period (1990–1999), moose were recorded in 61 squares also centered in the area of Třeboňsko PLA (Figure 4b), with regular occurrence and reproduction also identified near Lipno Reservoir, in the southern part of the Bohemian Forest Ecosystem. During the third period (2000–2009), moose occurrence was recorded in 71 squares. The core area of moose distribution shifted from Třeboňsko PLA to the southern part of the Bohemian Forest Ecosystem, along the Austrian border, and mostly consisted of regular occurrences and reproduction. There were two core areas of moose distribution (Figure 4c). During the last period (2010–2019), moose observations were registered in 66 squares, with the core areas of distribution fragmented into smaller patches along the Czech–Austrian border, especially near Lipno Reservoir. Moose reproduction was also recorded in the Novohradské hory and Třeboňsko PLA (Figure 4d).

### 3.2 Mortality

Between 1958 and 2019, there were 27 documented moose mortality events (see figure and table in Appendix 1, 2). Wildlife‐vehicle collisions accounted for the largest proportion (*n* = 13; 48.2%), followed by legal culling (3; 11.1%) and poaching (1; 3.7%). For the other 10 individuals (37.0%), it was not possible to determine the cause of death. The spatio‐temporal distribution of moose mortality is shown in Appendix 1, 2. Vehicle collisions resulting in moose deaths mainly occurred on minor roads (11 from 13 collisions, 84.6%); only two fatal collisions took place on primary roads and highways. The two moose died between 1989 and 1991 due to poaching/legal culling had been recorded in Austria (*n* = 2); one moose had been shot after hit by a car in Germany.

### 3.3 Habitat preferences

The AUC calculated for the habitat suitability model was 0.726, suggesting a fair fit (Araujo et al., 2005). Altitude contributed most to the model performance, followed by land cover, slope, and distance to human settlements (Table 2).

**TABLE 2:** Contribution of the variables to the model

Variable	Contribution (%)	Permutation importance
DEM (m a.s.l.)	54.7	59.1
Land cover (categorical)	23.4	8.2
Slope (°)	9.2	14.2
Distance to settlement (m)	7.9	6.3
Distance to road (m)	2.3	6.3
Road density (km/km^2^)	2	5
Distance to forest (m)	0.4	0.9

**FIGURE 6:**
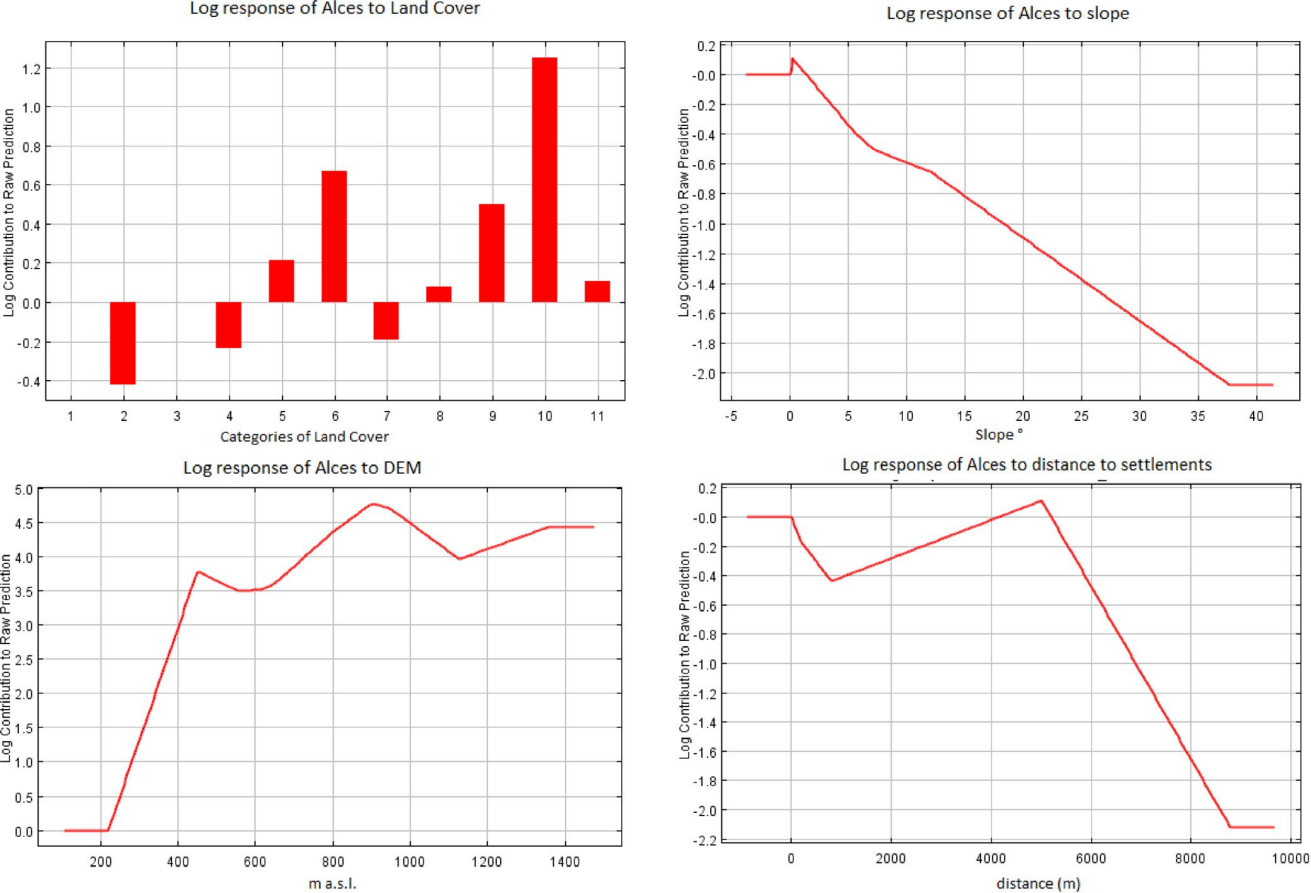
Response curves of the most important contributors in the Maxent habitat model. Land cover categories: (1) artificial surfaces, (2) arable land, (3) permanent crops, (4) pastures, (5) heterogeneous agricultural areas, (6) broad‐leaved forest, (7) coniferous forest, (8) mixed forest, (9) natural grassland and shrubs, (10) wetlands, and (11) waterbodies

The response curves of the different variables are shown in Figure 6. Altitudes above approximately 700 m were linked to a higher suitability. The habitat model predicted a higher suitability of wetland, broad‐leaved forest, natural grasslands, and shrubland, while arable land, pastures and coniferous forest and steep areas were of lower habitat suitability.

**FIGURE 7:**
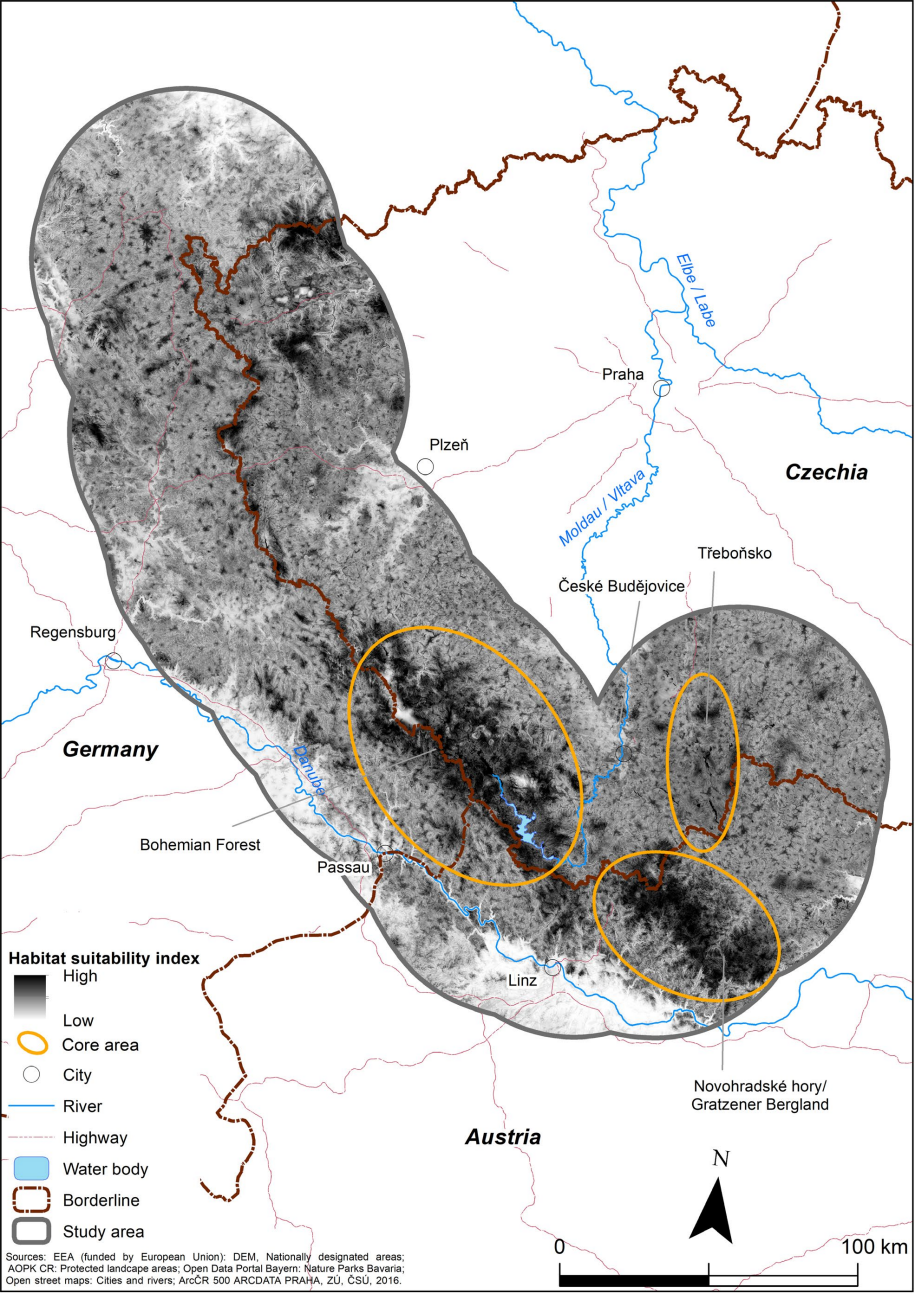
Results of the habitat suitability model based on Maxent modeling.

Human activity was represented by the distance to roads, the distance to settlements and road density. Habitat suitability was highest at intermediate distances to roads, with a slight decrease in the first few hundred meters, followed by an increase in suitability up to 3,000 m away from roads. The shape of the curve was similar to that of the distance from settlements, which were avoided within the first 1,000 m. Moose avoided areas with a high road density as well. The results of the habitat suitability model showed that suitable moose habitats within the study area were located in three subareas: (a) Třeboňsko (ca. 500 km^2^), (b) Bohemian Forest Ecosystem (ca. 2,000 km^2^), especially in the southern SNP and the Šumava PLA near Lipno Reservoir as well as at the transboundary in the eastern part of Bavaria and Austria, and (c) Novohradské hory/Gratzener Bergland/Freiwald/Weinsberger Wald (ca. 900 km^2^), especially the Austrian part (see Figure 7). Smaller patches of suitable habitat were dispersed at higher altitudes in the western and northern part of the study area, along the Czech–Bavarian border.

The validation with C1 and C2 only data provides similar results and as to expect a higher AUC 0.826 (see Appendix 3 for model). The habitat models for the distinct periods revealed for the first two periods (1958–1989 and 1990–1999) that preferred habitat tended to be at lower altitude with wetlands and water bodies. In contrast, in the periods of 2000–2009 and 2010–2019, suitable habitat shifted to higher altitudes, which also was the most important predictor (see Appendix 4).

In analysis of the distances from moose records to key land cover categories, we used the nonparametric Kruskal–Wallis test, because of non‐normal distributed data. Distances from the artificial surfaces are not significantly different across the periods (*p* = 0.051); however, the distance of the moose from artificial surfaces has been increasing from the second period (1990–1999; mean distance (m) 1958–1989 = 1,906, 1990–1999 = 1,826, 2000–2009 = 2,063, and 2010–2019 = 2,217). We also found that the distance of observations to wetlands (*p* = 0.000; mean distance (m) 1958–1989 = 11,590, 1990–1999 = 6,597, 2000–2009 = 10,746, and 2010–2019 = 9,968) and waterbodies (*p* = 0.000; mean distance (m) 1958–1989 = 6,516, 1990–1999 = 3,963, 2000–2009 = 5,663, and 2010–2019 = 6,922) increased while distance to broad‐leaved forest (*p* = 0.008; mean distance (m) 1958–1989 = 6,033, 1990–1999 = 6,369, 2000–2009 = 6,106, and 2010–2019 = 4,219) and natural grassland and shrubs (*p* = 0.000; mean distance (m) 1958–1989 = 2,713, 1990–1999 = 4,369, 2000–2009 = 4,069, and 2010–2019 = 3,066) decreased from 1990s.

## 4 DISCUSSION

Our study provides the first comprehensive assessment of the long‐term development of occurrence, mortality, and habitat association of the moose population at the southernmost edge of its range in Europe. Specifically, habitat suitability (Krausman, 1999) and causes of mortality were evaluated using available observational data covering the period between 1958 and 2019. Our results showed that for many years the number of moose observations increased, peaking in the 1980s, the 1990s, and again around 2010 (Figure 5); however, there was a steep decline after 2013. During the third period (2000–2009), the core area of moose distribution shifted from Třeboňsko to the southern SNP and Šumava PLA. The main causes of reported moose mortality were human‐related, especially vehicle collisions, which increased in the fourth period (2010–2019). Our habitat model showed the availability of sufficient suitable moose habitat, identified as areas far from settlements and with low road densities, and especially flat areas at higher altitudes containing broad‐leaved forests, wetlands, and open landscapes (i.e., natural grasslands with shrubs).

The first records of moose occurrence in our study area were from what is now the Czech Republic (1958, although the first record from the Czechia was from 1957; Červený et al., 2001), followed by Austria (1964) and Germany (1976) (Schönfeld, 2009), reflecting a dispersal from Poland (Tomek, 1977). As there was no systematic monitoring of moose during the study period, the factors contributing to the inconsistent number of observations independent of moose density remain unclear. Whether the intensity of moose monitoring has differed across the decades or people's awareness of the animals has changed is unknown, as no information about the search efforts was available. Furthermore, some records were gathered by citizen scientists, which can lead to a spatially and temporal uneven coverage of the very large study area and also incorrect observations (Geldmann et al., 2016). However, the evaluation of our results using only verified data (C1 and C2) revealed similar results and underlined the reliability of the data collected by citizen scientists. While an intensification of monitoring may have contributed to the increase in moose observations in the 2000s, it does not explain the steep decrease of the observation in the most recent years. The current population size cannot be estimated precisely with the available data, but published estimates from the early 1990s of up to 25 individuals in Třeboňsko and 10–20 individuals in the SNP’s Lipno Reservoir contrast with estimates from 2010 for the same localities: 0 and 10–15 individuals, respectively (Mrlík, 1995; Romportl et al., 2017). The reported extinction of the species in Třeboňsko in 2010 (Romportl et al., 2017) seems to have been premature, as post‐2010 records show moose re‐expansion, including that of young animals (Figure 4d). Nonetheless, although the number of recorded observations was highest during the fourth period, according to local experts the reduced number of observations since 2013 and the small patches of occurrence indicate a population decline. This negative trend is contrary to both the population increase of ungulates in Europe, including in the study region (Appolonio et al., 2010; Heurich et al., 2010; Linnel et al., 2020), and the increase in the moose population in neighboring Poland, with about 1,500 individuals recorded at the beginning of the 2000s and 28,000 in 2016 (Borowik et al., 2018). However, according to the authors of the latter study, this may be an overestimate by up to 46%, due to the limited reliability of the methods used to estimate abundance, such as drive counts, aerial surveys, and distance sampling (Bobek et al., 2013; Dziki‐Michalska et al., 2019). Regardless of the exact magnitude, the positive population growth in Poland is in contrast to the apparent population decline suggested by the observation data from our study area. This decline in the size and dispersal of the moose population cannot be explained by low habitat suitability; a more plausible explanation is the combined effects of mortality resulting from vehicle collisions and, perhaps, poaching. Moreover, the moose population in our study area was disjunctive, separated by distances of ca. 300 km and by densely populated landscapes, unlike the continuous species range in Poland. Importantly, the highway section of the motorway A4 between Krakow and the Ukrainian border, built between 2010 and 2016, follows almost exactly the southern border of the continuous range of moose in Poland, thereby severely limiting moose migration to the south (similarly in Czechia and motorway D1, Strnad et al., 2012). This fragmentation effect of motorways is also limiting dispersal to areas with suitable habitat in the western and northern part of our study area. A further consequence is a severe limitation of gene flow between moose in the study area and the source population in Poland. Research from Norway demonstrated the strong effects of environmental stochasticity on moose survival, especially that of calves, and the strong seasonal or regional variation acting upon the survival of adult females (Stubsjøen et al., 2010). Thus, another important factor for the limited population growth may be the vulnerability of the moose population to stochastic events. This might be especially relevant for our study site, as stochastic variation, including environmental and demographic stochasticity, is much higher for small, isolated populations.

Besides the reduction in moose observations, the spatial distribution of the population changed. The core area of moose distribution was initially in the Třeboňsko area but after 2000 it shifted to the southern part of the Bohemian Forest Ecosystem. Between 2010 and 2019, the population was dispersed around the Lipno Reservoir, with occurrences in Třeboňsko PLA restricted to small patches. The shift from Třeboňsko PLA can be attributed to the high level of human activities in this area (e.g., higher human population density, traffic intensity, and recreational activities) than in the Bohemian Forest Ecosystem, which corresponded with increasing distance to artificial surfaces and can be caused by increased human disturbance (e.g., recreational activities) leading to less suitable habitat. Moreover, the Třeboňsko PLA is located at lower elevations, where temperatures are higher, in contrast to the lower temperatures in the Bohemian Forest Ecosystem due to its higher elevation, on the other hand wetlands and water bodies that can be used to mitigate heat stress are more presented in Třeboňsko and distances of the moose from this landscape features have been increasing since 1990s (Beest et al., 2012; Bjørneraas et al., 2011). Recent studies have shown an increase in the temperatures at the study site (CHMI, 2018; Fick & Hijmans, 2017), such that the temperatures of 14°C in summer and −5°C in winter, the upper thresholds for the well‐being of moose (Beest et al., 2012; Bjørneraas et al., 2011), may be regularly exceeded. The mean temperatures in the areas of Poland harboring larger moose populations are generally lower than in Třeboňsko PLA (Borowik et al., 2018). Therefore, a contribution of the changing climate, alone or in combination with anthropogenic pressure (traffic, recreation), to the decline in moose population size in the study area, particularly in Třeboňsko, cannot be ruled out (Beest et al., 2012; Bjørneraas et al., 2011).

Humans accounted for 64.3% of the recorded deaths in the studied moose population, with vehicle collisions playing the largest role and increasing between 2010 and 2019 (Andreska, 2017). Vehicle collisions were also an important cause of moose mortality in previous studies conducted in other moose populations (Neumann et al., 2012; Sailer, 2005). The majority of the collisions documented in our study occurred on low‐traffic roads, especially those around the Lipno Reservoir. Sailer (2005) similarly found the highest probability of moose‐vehicle collisions on roads with intermediate traffic volumes (see also Dussault et al., 2006; Rea, 2003). This suggests that moose avoid major and international roads, due to their high traffic intensity and the associated noise and light pollution, both of which discourage moose crossings (Niemi et al., 2017). Our habitat suitability models likewise showed that moose avoid roads. However, considering their large home ranges and dispersal distances, moose will inevitably seek to crossroads and will thus be at risk of being struck by vehicles.

Human‐caused mortality, including legally culled and poached animals, was the second most important cause of moose mortality, despite the fact that moose are fully protected by nature conservation and/or hunting laws in Austria, the Czech Republic, and in Germany. Only in Austria, moose can be shot, but only in exceptional cases, such as when their intense browsing damages young forests (Mrlík, 1995). Although just one moose was confirmed as poached in 1989, undetected illegal killing may have occurred throughout the study area, as previously demonstrated for other protected species in the same region (Heurich et al., 2018). Long‐term studies in Scandinavia have shown that variations in human‐induced mortality (legal culling, poaching) have substantial effects on moose population dynamics (Solberg et al., 1999). In Poland, hunting and poaching had a strong impact on moose populations, but a 2001 ban on moose hunting resulted in their steady increase (Borowik et al., 2018; Bragina et al., 2018; Tomek, 1977) to the extent that wildlife managers suggested the re‐introduction of culling (Dziki‐Michalska et al., 2019). Predation and diseases are leading causes of moose mortality (Okarma et al., 1995; Severud et al., 2019) but there were no reports of diseases and/or severe infections among the moose in the study area, possibly because of the low population density. Predation was also not recorded in the study area, as there have been no bears in the study area and wolf recolonization just occurred recently.

Modeling habitat suitability from observation data is based on several assumptions. The first, and most important, is that there is no observation bias. As moose are obviously easily determined to the species level, we are confident that the sampling coverage of our study area was good and that the observers were highly willing to report observations. In addition, our results are in agreement with other studies of moose habitat selection (Beest et al., 2012; Borowik et al., 2018, 2020; Melin et al., 2016; Romportl et al., 2017), such that the potential bias was probably low. Thus, based on the results of our habitat model, the ideal habitat for moose in the studied area is a mosaic of forest, to provide shelter, more open extensive landscape with natural succession, as a source of food, and water bodies and wetlands for mitigating heat stress (Bjørneraas et al., 2011; Beest et al., 2012). Although this overall landscape preference is similar to moose habitats in Poland (Borowik et al., 2018; Tomek, 1977), within our study area these combined features occur at markedly higher altitudes (700–1,000 m. a.s.l.). In fact, during the study period, there was a shift from lower to higher altitudes that might have been induced by the increasing temperatures and resulting heat stress (Beest et al., 2012; Bjørneraas et al., 2011). The smaller peak in the response curve between 400 and 500 m a. s. l. represents the increased of moose occurrence in the flat landscape of Třeboňsko before 2000. However, after 2000, Třeboňsko was less often frequented by moose despite the large number of fishponds and wetlands offering relief from the hot summers and the abundance of woody plant communities dominated by the taxa preferred by moose for browsing (*Salix* spp., *Prunus padus,* etc.). The decline in moose observations at Třeboňsko coincided with the increasing avoidance of the artificial surfaces and an enormous increase in recreational activities over the last three decades, including biking, hiking, camping, and canoeing, such that this previously relatively undisturbed area has become intensely used by humans throughout the vegetation period (Klufová et al., 2006). The stress related to human presence may be an overlooked but significant factor inducing a preference shift from otherwise suitable habitat, in this case from Třeboňsko to the SNP, where there are still relatively large areas devoid of people. Nonetheless, between 2010 and 2019, the largest number of vehicle collisions occurred around the SNP’s Lipno Reservoir and in the Šumava PLA. Moose generally prefer flat terrain, which is linked with wetlands (mostly peat bogs) and water bodies in core areas of occurrence. Schönfeld (2009) found that moose occurrence in Bavaria was highest in more forested areas, and in Poland in large forest complexes (Tomek, 1977). Wetlands provide thermoregulation and forage and are thus the most favored land cover type for moose (Bjørneraas et al., 2011; Dussault et al., 2004); however, the distance of moose records to wetlands increased throughout the study period. In winter, coniferous forests offer better conditions for hiding and foraging (Bjørneraas et al., 2011) whereas in summer broad‐leaved forests and shrubs enable browsing (Månsson, 2009). The broad‐leaved forests in the study area provide better forage, whereas hiding in coniferous forests is not an absolute necessity. This would account for the larger contribution of broad‐leaved forests to the model's fit and still higher proximity of the moose records to this land cover category through the periods. For foraging, natural grassland and shrubs were also identified by the model as important habitat feature. This finding is in agreement with the conclusion of Schmölcke and Zachos (2005) that deforestation is not necessarily a negative factor as it also results in a rich food supply. However, in contrast to the findings of Romportl et al., (2017), pastures were not identified as preferred habitat in our model. These differences could reflect the different methodologies, because Romportl et al., (2017) merged meadows and pastures (according to Corine, pastures are more intensively used) into one class while in our study pastures, natural grasslands and heterogeneous agriculture areas were considered separate classes. As arable land is generally intensively used by humans, it is avoided by moose.

A shortcoming of our study is that our model does not provide a seasonal resolution, due to the limited data availability. However, it is suitable in predicting annual moose habitat, including that reached by seasonal migrations (Borowik et al., 2020). Sweanor and Sandegren (1988) determined a migration distance of 320 km in Sweden and migrations in Alaska of up to 280 km were reported by Gasaway et al., (1983). These long migrations may increase the risk of vehicle collisions (Tajchman et al., 2017). Thus, future research on moose should focus on their seasonal habitat selection and their long‐distance movements.

## 5 CONCLUSIONS

Our study showed a decrease in the moose population in recent years and a shift in its core area from Třeboňsko to a more fragmented distribution within smaller patches in the SNP, Šumava PLA, Novohradské hory, and Třeboňsko PLA. Young animals were seen in these patches between 2010 and 2019, but the area where calves were born and reared could not be determined. In agreement with other studies from Central Europe, our study showed that moose prefer all types of forest and more extensive types of open landscape, including (semi)natural grasslands, for foraging. However, flat terrain associated with wetlands and water bodies was the most favored habitat. Together with the observed shift of moose populations to higher elevations, this preference may reflect a strategy against heat stress and human‐caused disturbance, as also indicated by our habitat model. Another finding of our study was that the availability of suitable habitats is not a limiting factor for the survival of moose populations; rather, the greatest threat is increasing traffic (with increasing probability of moose‐vehicle collisions), which should be taken into account in future conservation measures. While illegal killings could not be evaluated with respect to the effects on moose population growth, the strong demographic stochasticity acting upon small populations suggests a substantial role. Other factors, including the rising temperatures in low‐lying areas resulting from climate change and the increase in recreational activities in areas preferred by moose, likely also contributed to the decline in the numbers of these animals at the southernmost edge of the study area.

Based on a comprehensive synthesis of available records gathered from three countries, this work is a first step toward a complete assessment of the moose population in the study area. Meanwhile, also samples for genetic analysis are being collected in all three countries. The next step would be to implement a systematic monitoring programme aimed at collecting more detailed information on the spatial‐temporal habitat requirements of moose as well as demographic data, such as population density, reproduction, survival, and genetic structure.

The moose population in our study area was isolated and very small, possibly less than 20 animals. Moreover, it seems to be decreasing, with inbreeding and the factors considered in this study likely to drive it further down the extinction vortex. Our results highlight the urgent need for mitigation measures to prevent moose‐vehicle collisions and illegal killings. Based on the low number of animals in the study area, it can be expected that active measures such as translocation are necessary for the long‐term viability of moose population in the study area. Given the large scale spatial requirements of these herbivores, coordinated cross‐border management aimed at the conservation of this population is essential.

## CONFLICT OF INTEREST

The authors have declared that no competing interests exist.

## AUTHOR CONTRIBUTION

**Tomáš Janík:** Conceptualization (lead); Data curation (lead); Formal analysis (lead); Funding acquisition (equal); Investigation (equal); Methodology (equal); Project administration (equal); Resources (equal); Software (lead); Supervision (supporting); Validation (equal); Visualization (lead); Writing‐original draft (lead); Writing‐review & editing (equal). **Wibke Peters:** Conceptualization (equal); Data curation (equal); Methodology (equal); Writing‐original draft (equal); Writing‐review & editing (equal). **Martin Salek:** Conceptualization (lead); Data curation (equal); Methodology (lead); Validation (equal); Writing‐original draft (equal); Writing‐review & editing (lead). **Dušan Romportl:** Conceptualization (supporting); Data curation (supporting); Investigation (equal); Methodology (equal); Writing‐original draft (equal); Writing‐review & editing (equal). **Miloslav Jirků:** Conceptualization (equal); Data curation (equal); Formal analysis (equal); Funding acquisition (equal); Writing‐original draft (equal); Writing‐review & editing (lead). **Thomas Engleder:** Data curation (equal); Writing‐original draft (equal); Writing‐review & editing (equal). **Martin Ernst:** Data curation (equal); Investigation (equal); Writing‐original draft (supporting). **Jiří Neudert:** Data curation (equal); Writing‐original draft (equal). **Marco Heurich:** Conceptualization (equal); Data curation (equal); Formal analysis (supporting); Investigation (equal); Methodology (supporting); Project administration (supporting); Resources (equal); Supervision (lead); Validation (equal); Writing‐original draft (equal); Writing‐review & editing (equal).

## Supporting information

Appendix S1

Appendix S2

Appendix S3

Appendix S4

Supplementary Material

## Data Availability

Dataset is available on DRYAD https://doi.org/10.5061/dryad.z612jm69s.
