# Supplementary figures and images for: The declining occurrence of moose (Alces alces) at the southernmost edge of its range raise conservation concerns
Source: Ecol Evol. 2021 Mar 30;11(10):5468–83. doi: 10.1002/ece3.7441 (PMC8131793; doi:10.1002/ece3.7441)

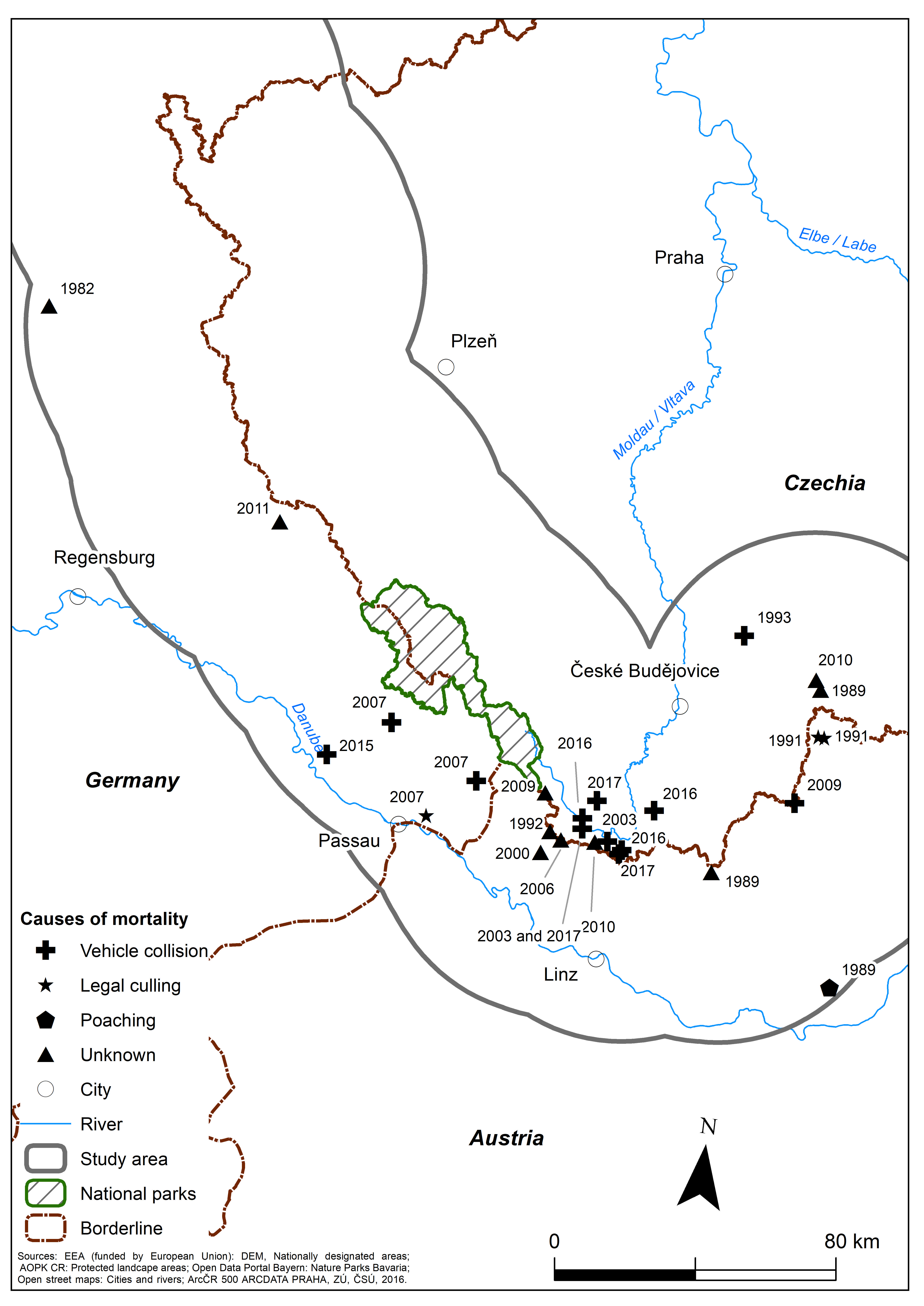

Supplement: Supplementary file 1 — Appendix S1 [file ECE3-11-5468-s001.tif]

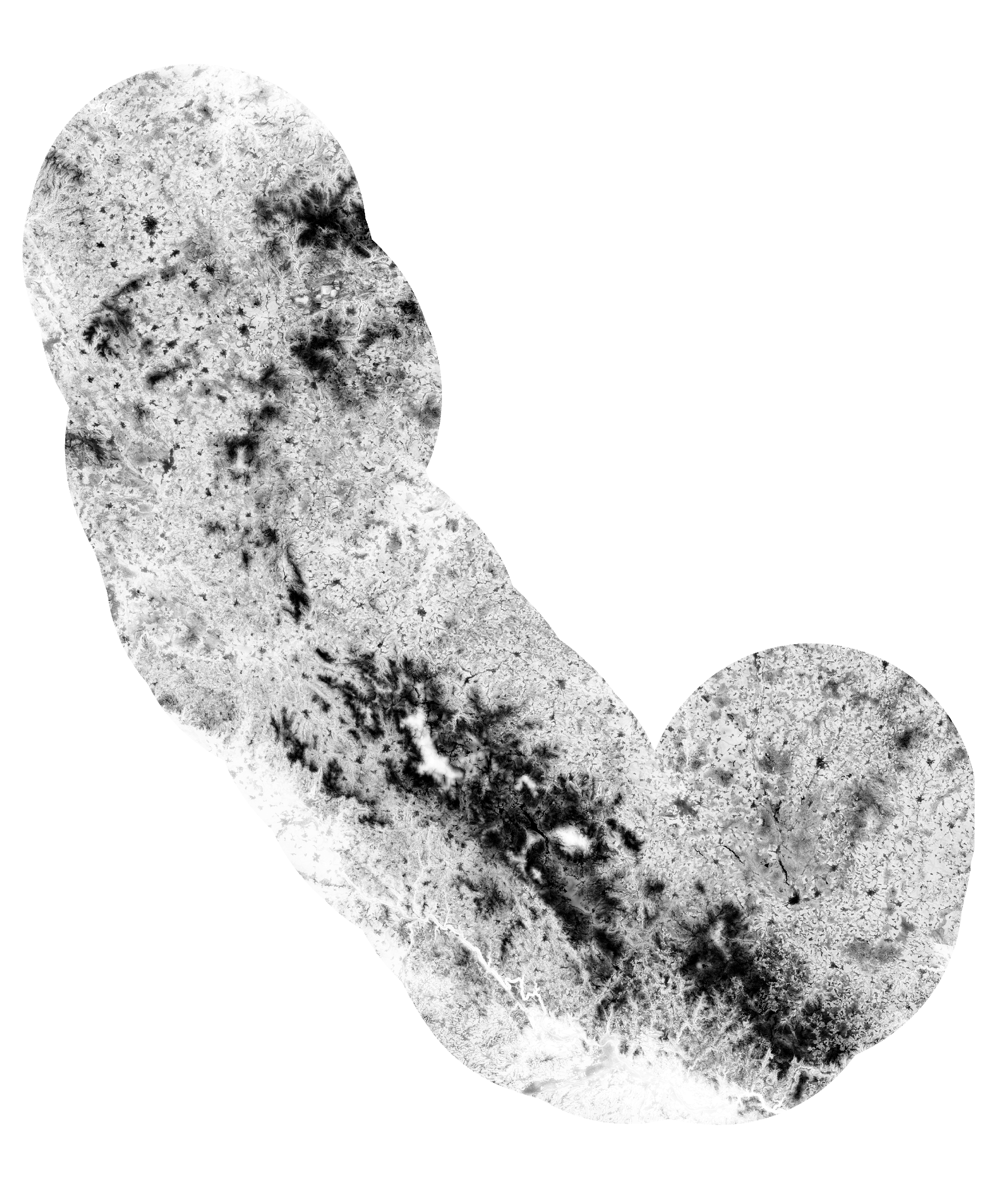

Supplement: Supplementary file 3 — Appendix S3 [file ECE3-11-5468-s005.tif]

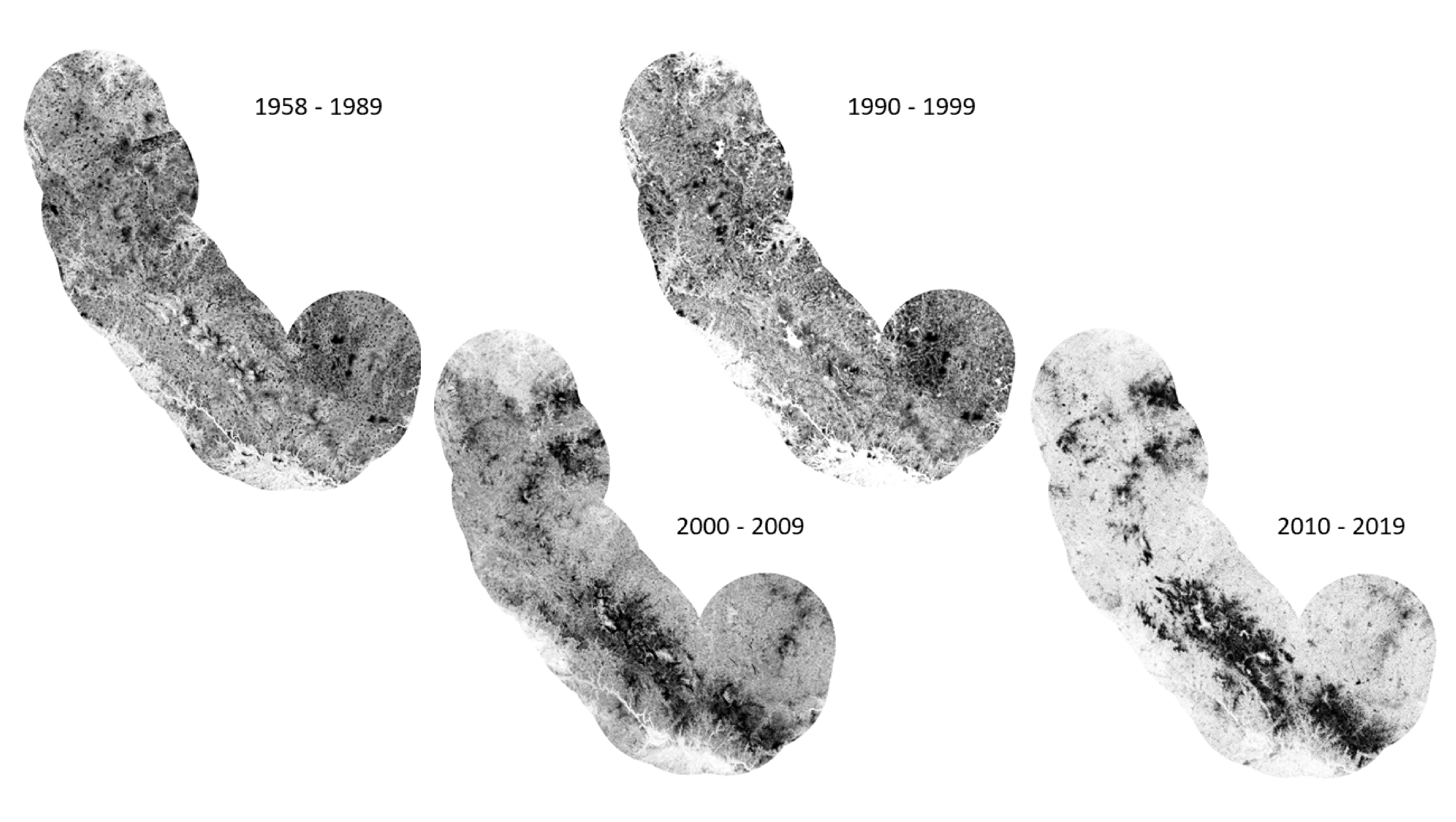

Supplement: Supplementary file 4 — Appendix S4 [file ECE3-11-5468-s004.tif]
