# Supplementary material for: The declining occurrence of moose (Alces alces) at the southernmost edge of its range raise conservation concerns
Source: Ecol Evol. 2021 Mar 30;11(10):5468–83. doi: 10.1002/ece3.7441 (PMC8131793; doi:10.1002/ece3.7441)
Supplement: Supplementary file 2 — Appendix S2 [file ECE3-11-5468-s002.docx]

| **Period** | **Vehicle collisions** | **Legal culling** | **Poaching** | **Unknown** |
| --- | --- | --- | --- | --- |
| **1958–1989** | 0 | 0 | 1 | 3 |
| **1990–1999** | 1 | 2 | 0 | 1 |
| **2000–2009** | 5 | 1 | 0 | 3 |
| **2010–2019** | 7 | 0 | 0 | 3 |

Appendix 2: Moose mortality records in the study area during the four periods of the study.
