# Supplementary material for: The declining occurrence of moose (Alces alces) at the southernmost edge of its range raise conservation concerns
Source: Ecol Evol. 2021 Mar 30;11(10):5468–83. doi: 10.1002/ece3.7441 (PMC8131793; doi:10.1002/ece3.7441)
Supplement: Supplementary file 5 — Supplementary Material [file ECE3-11-5468-s003.docx]

**Appendix: mortality**

Appendix 1: Spatial-temporal distribution of moose mortality in the study area, 1958 – 2019 (figure).

Appendix 2: Moose mortality records in the study area during the four periods of the study (table).

**Appendix: Habitat suitability models (black = higher suitability, white = lower suitability)**

Appendix 3: Verification of the model by C1 and C2 data only with the same environmental variables. Results gave with higher AUC 0.826 similar results (=similar key areas of suitable habitat and contribution of the predictors) to the general model (see Fig. 6,7).

Appendix 4: To illustrate the development of the distribution and its suitability during the periods, we performed the model for each period. It shows shift from the lower altitude to higher (from Třeboňsko to Bohemian Forest Ecosystem). We performed these models based on records for each period and with the same environmental variables except land cover (we used mentioned temporal horizons of the CLC). We think it is sufficient for demonstration of distribution development. Moreover, surface characteristics did not change. Roads and settlement have changed but they are smaller contributors in the model.
